# Spatial Distribution and Predictive Significance of Dendritic Cells and Macrophages in Esophageal Cancer Treated With Combined Chemoradiotherapy and PD-1 Blockade

**DOI:** 10.3389/fimmu.2021.786429

**Published:** 2022-01-03

**Authors:** Xiaoxue Ma, Zhoubo Guo, Xiaoying Wei, Gang Zhao, Dong Han, Tian Zhang, Xi Chen, Fuliang Cao, Jie Dong, Lujun Zhao, Zhiyong Yuan, Ping Wang, Qingsong Pang, Cihui Yan, Wencheng Zhang

**Affiliations:** ^1^ Department of Radiation Oncology, Tianjin Medical University Cancer Institute and Hospital, National Clinical Research Center for Cancer, Key Laboratory of Cancer Prevention and Therapy, Tianjin’s Clinical Research Center for Cancer, Tianjin, China; ^2^ Department of Pathology, Tianjin Medical University Cancer Institute and Hospital, National Clinical Research Center for Cancer, Key Laboratory of Cancer Prevention and Therapy, Tianjin’s Clinical Research Center for Cancer, Tianjin, China; ^3^ Department of Endoscopy Diagnosis and Therapy, Tianjin Medical University Cancer Institute and Hospital, National Clinical Research Center for Cancer, Key Laboratory of Cancer Prevention and Therapy, Tianjin’s Clinical Research Center for Cancer, Tianjin, China; ^4^ Department of Nutrition Therapy, Tianjin Medical University Cancer Institute and Hospital, National Clinical Research Center for Cancer, Key Laboratory of Cancer Prevention and Therapy, Tianjin’s Clinical Research Center for Cancer, Tianjin, China; ^5^ Department of Immunology, Tianjin Medical University Cancer Institute and Hospital, National Clinical Research Center for Cancer, Key Laboratory of Cancer Immunology and Biotherapy, Tianjin’s Clinical Research Center for Cancer, Tianjin, China

**Keywords:** chemoradiotherapy, PD-1, esophageal cancer, dendritic cell, macrophage, spatial, immunofluorescence, tumor mutation burden

## Abstract

**Background:**

The first clinical study (NCT03671265) of first-line chemoradiotherapy combined with PD-1 blockade showed promising treatment outcomes in locally advanced esophageal squamous cell carcinoma (ESCC). However, partial patients did not respond to the combination treatment. The roles of dendritic cells (DCs) and macrophages in this combination treatment remain poorly understood.

**Methods:**

We performed multiplexed immunofluorescence method to identify CD11c^+^ DCs, CD68^+^ macrophages, and their PD-L1^-^ or PD-L1^+^ subpopulations in paired tumor biopsies (*n* = 36) collected at baseline and during the combination treatment (after radiation, 40 Gy) from the phase Ib trial (NCT03671265). We applied whole exome sequencing in the baseline tumor biopsies (*n* = 14) to estimate tumor mutation burden (TMB). We dynamically investigated the spatial distribution of DCs and macrophages under chemoradiotherapy combined with PD-1 blockade, and evaluated the association between their spatial distribution and combination outcome, and TMB.

**Results:**

The results showed that high percentages of PD-L1^-^ DCs and macrophages in the baseline tumor compartment, but not in the stromal compartment, predicted improved OS and PFS. Chemoradiotherapy combined with PD-1 blockade promoted DCs and macrophages to migrate closer to tumor cells. During combination treatment, PD-L1^-^ tumor cells were nearest to PD-L1^-^ DCs and macrophages, while PD-L1^+^ tumor cells were next to PD-L1^+^ DCs and macrophages. High TMB was closely associated with a shorter distance from tumor cells to DCs and macrophages. Shorter distance between PD-L1^+^ tumor cells and PD-L1^+^ DCs or PD-L1^-^ macrophages during the combination was correlated with better OS. Shorter distance between PD-L1^-^ tumor cells and PD-L1^-^ macrophages during combination was associated with both longer OS and PFS.

**Conclusions:**

PD-L1^-^ or PD-L1^+^ DCs and macrophages exhibit distinct spatial distribution in ESCC. The close distance between tumor cells and these antigen-presenting cells (APCs) is critical to the clinical outcome in chemoradiotherapy combined with PD-1 blockade in ESCC patients. Our results highlight the predictive potential of spatial patterns of APCs in chemoradiotherapy combined with immunotherapy and reveal the underlying mechanism of APCs participating in chemoradiotherapy-induced antitumor immune response in ESCC.

## Introduction

Chemoradiotherapy induces immunogenic cell death and triggers antitumor immunity (1). Recent clinical studies demonstrated that combining chemoradiotherapy with PD-1 blockade as first-line treatment had promising therapeutic efficacy in locally advanced solid tumors beyond esophageal squamous cell carcinoma (ESCC) (2, 3). We first conducted a clinical trial of first-line chemoradiotherapy combined with anti-PD-1 antibody camrelizumab in locally advanced ESCC. A total of 65% patients survived for at least 2 years, but partial patients did not benefit from this combination (4). It urgently needs to identify potential biomarkers in patients treated with chemoradiotherapy combined with PD-1 blockade.

High tumor-infiltrating lymphocytes (TILs) were associated with improved survival in patients receiving definitive chemoradiotherapy (5). Patients with high CD8^+^/Foxp3^+^ T-cell ratio had favorable survival after surgery (6, 7). In addition to T cells, tumor-infiltrating dendritic cells (DCs) and macrophages played important roles in the initiation and regulation of innate and adaptive antitumor immune response in multiple tumors (8, 9). Their antitumor effect can be attributed to their antigen-presenting function, and they are called antigen-presenting cells (APCs). APCs promoted antitumor immunity or tolerance by presenting antigens to T cells and providing immunomodulatory signals through cell–cell contact and cytokines after sensing the changes from tumor cells and the microenvironment (10, 11). PD-L1 expression on DCs and macrophages attenuated T-cell activation and induced tumor escape (12, 13). Chemoradiotherapy could convert “cold” tumors to “hot” tumors by the evidence of elevated CD8^+^ T cells, DCs, and macrophages in tumor microenvironment (14, 15). However, the alteration of DCs and macrophages under chemoradiotherapy combined with immunotherapy and its association with treatment outcome is little known in ESCC.

Although accumulated studies reveal that the composition of tumor-infiltrating immune cells is important to antitumor immune response, most previous studies did not consider the reciprocal interaction between immune cells and tumor cells. The distribution of immune cells in the tumor microenvironment presented spatial distinction and subset-specific prognostic significance (16, 17), which might identify the potential mechanisms of the antitumor immune response.

In the present study, we collected paired tumor biopsy samples from the phase Ib clinical trial of chemoradiotherapy combined with anti-PD-1 antibody camrelizumab as the first-line therapy in locally advanced ESCC (ClinicalTrials.gov NCT03671265) at baseline and during combination (after radiation, 40 Gy) (4). We prospectively identified the DCs and macrophages in the tumor microenvironment to illustrate the dynamic spatial location of DCs and macrophages responding to chemoradiotherapy combined with PD-1 blockade, which provides predictive candidates for clinical outcome of the combination in ESCC.

## Methods

### Study Design and Sample Collection

The phase Ib study evaluating the safety and feasibility of definitive chemoradiotherapy combined with an anti-PD-1 antibody, camrelizumab, as the first-line therapy in locally advanced ESCC (ClinicalTrials.gov NCT03671265) (4). Specifically, camrelizumab (SHR1210, Jiangsu Hengrui Medicine Co. Ltd., China) was given on day 1 of every 2-week period from the beginning of radiotherapy up to 32 weeks, concurrently with radiotherapy for 6 weeks, and with chemotherapy for 4 weeks (4). The exploratory endpoints of this phase Ib study were local and systematical immune characteristics, and potential predictive biomarkers for combination treatment outcome.

Baseline (*n* = 20) and on-treatment (after 40 Gy radiation, *n* = 18) tumor biopsies were collected (Additional file 1: **Table S1**). Deep biopsy samples of tumor tissues were collected under endoscopic ultrasonographic guidance (18, 19) and made into formalin-fixed paraffin-embedded (FFPE) tissue blocks.

### Ethics Statement

This study was conformed to the ethical principles outlined in the Declaration of Helsinki, and the protocol was approved by the institutional review board and ethics committee at Tianjin Medical University Cancer Institute & Hospital (E2018142). All patients provided written informed consent to participate. This study was registered on ClinicalTrials.gov (NCT03671265).

### Multiplex Immunofluorescence Staining

To dynamically monitor the tumor immune microenvironment at baseline and during the combination, serial FFPE slides of the biopsy specimens were stained by using tyramide signal amplification (TSA)-based multiplex immunofluorescence assay method. The multi-color immunofluorescence staining was automatically performed in Bond III automated stainer (Leica, USA). The TSA 5-color kit (#D110051-50T) and TSA 670 (#D110016-100T) were bought from Yuanxibio, China. The stanning panel was as follows: Anti-PD-L1 (#13684, CST, 1:800)/TSA 570, anti-panCK (#GM351507, Gene Tech, 1:6)/TSA 520, anti-CD11c (#45581, CST, 1:300)/TSA 620, anti-CD68 (#GM087602, Gene Tech, ready-to-use)/TSA 670. In the first staining cycle, FFPE slides were immersed in xylene to remove paraffins on the slides. Transfer slides to 100%, 95%, 70%, and 50% alcohol, respectively. Perform antigen retrieval to unmask the antigenic epitope by using microwave treatment in optimal buffer as recommended. Add blocking buffer onto the sides. Drain off blocking buffer from the slides and apply appropriately diluted primary antibody. Add HRP-conjugated second antibody. Then, add fluorescent TSA reagent. Microwave treatment was applied to remove the first antibodies deposited and the staining process is repeated for a subsequent target. The process is repeated until all targets have been labeled. In the last steps before imaging, add 4’,6-diamidino-2-phenylindole (D1306; Thermofisher) to visualize cell nuclei and apply a cover slip. The slides were ready to image.

### Imaging and Analysis

A whole slide scan was performed for each fluorescence-stained slide using a digital microscopy scanner Pannoramic MIDI tissue imaging system (3DHISTECH Ltd., Hungary). Because both tumor cells and normal epithelial cells have positive CK expression, it is hard to distinguish these two cell types in immunofluorescence staining. To exclude the normal epithelial cells in analysis, we applied Hematoxylin and Eosin (H&E) staining in the tissue sections after finishing the fluorescence scan. Images were analyzed by Indica Halo software (Indica Labs, UK). Two independent blinded pathologists performed histologic evaluation and supervised to split the tumor and stromal compartments by using Halo software. Cells were phenotyped into the following subsets: DC (CD11c^+^), macrophage (CD68^+^), tumor cell (CK^+^), and PD-L1^+^ subpopulations of these cells.

Immune cell infiltration was evaluated as the number of cells per slide, in the tumor compartment, stromal compartment, or total viable tissue area of the slides, respectively. To evaluate the spatial relationship between immune cells and tumor cells, the distance between each tumor cell and its nearest neighbor immune cells was measured.

### Tumor Mutation Burden Test

To investigate the tumor mutation at baseline, the biopsy specimens from 14 patients before the combination were sequenced by using FoundationOne CDx (F1CDx) and FDA-approved 324-gene panel assay conducted by DIAN (Hangzhou Lab) with licensed technologies, to assess the tumor mutation burden (TMB) (4, 20).

### Statistical Analyses

Statistical significance between groups was compared using non-parametric two-sided Mann–Whitney *U* tests for two independent samples or Wilcoxon Signed-Rank tests for paired samples, and correlations were evaluated assuming a non-Gaussian distribution (Spearman correlation) unless otherwise indicated. OS was defined as the time from inclusion until death from any cause or the last date of follow-up time. Progression-free survival (PFS) was defined as the time from inclusion until the date of objective disease progression or death from any cause in the absence of progression. The Kaplan–Meier analysis was used to estimate OS and PFS. Differences in survival were compared with log-rank tests. The best cutoff of Kaplan–Meier survival analysis was calculated by the Youden index of the ROC curve.

All analyses were performed using SPSS v.25.0 (STATA, College Station, TX, USA). Reported *p* values were two-sided, and the significance level was set at 0.05. Survival curves and summary graphs were performed using GraphPad Prism v.8.0. The data cutoff date for all analyses was May 1, 2021.

## Results

### DCs and Macrophages in the Tumor Compartment Associated With Improved Survival

**Figure 1:**
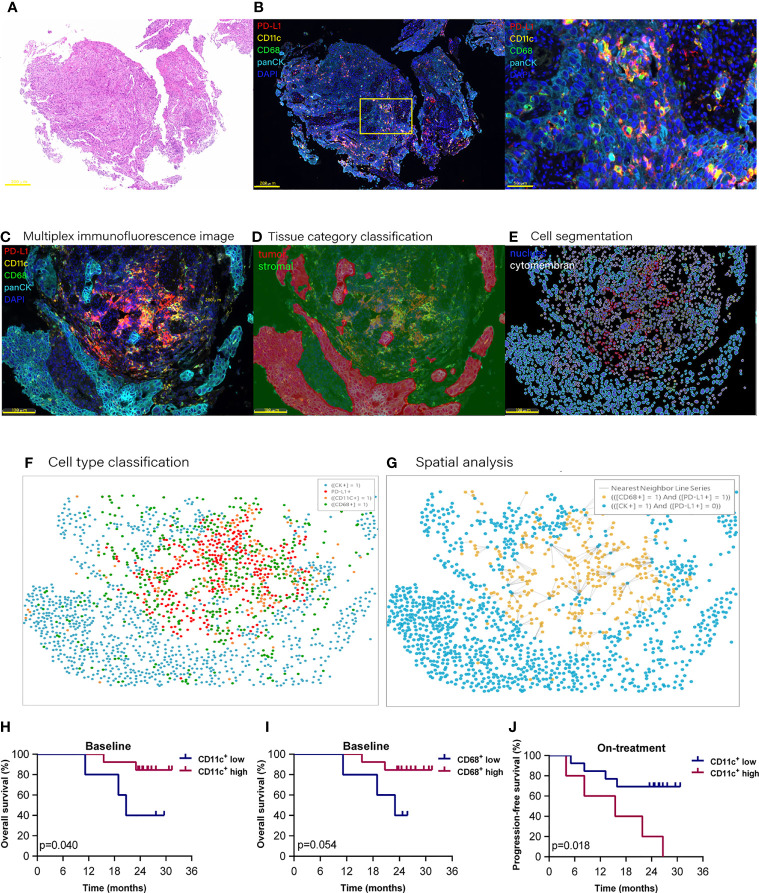
Proportion of dendritic cells and macrophages in the tumor compartment associated with improved survival. Hematoxylin and eosin staining **(A)** and multiplex immunofluorescence staining **(B)** for dendritic cells and macrophages in a tissue section (case *n* = 14, before treatment). **(B)** Right, Enlarged area of the yellow frame in left. **(D–G)** Spatial analysis procedure (case *n* = 6, before treatment). Kaplan–Meier curves showing overall or progression-free survival of ESCC patients based on the proportion of dendritic cells or macrophages in **(H, I)** the baseline tumor compartment and **(J)** on-treatment stromal compartment. Cutoff value: **(H)** 2.987%; **(I)** 1.623%; **(J)** 22.362%. On-treatment, after 40 Gy radiation. *p* ≤ 0.05, statistically significant.

We used multiplex immunofluorescence to identify DCs and macrophages in the tumor microenvironment (**Figures 1A–G**). A total of 36 scanned slides were finally included in analysis except for two baseline slides without tumor tissues, including 18 baseline and 18 on-treatment specimens, with 16 matched pairs at these two time points (Additional file 1: **Table S1**).

At the updated data cutoff date of May 1, 2021, the median follow-up duration was 26.6 months (95% CI 24.3 to 29.0). Thirteen patients were alive, and 11 patients were free of progressive disease (Additional file 1: **Table S2**). The OS and PFS ranged from 8.2 to 31.4 months and from 3.9 to 31.4 months, respectively. We initially analyzed the association between the total CD11c^+^ DCs, CD68^+^ macrophages (included in both tumor and stromal compartments), and the clinical outcome. However, the results of Kaplan–Meier analysis showed that neither the total DCs nor the total macrophages were associated with patient survival.

To investigate if these APCs located in different tissue compartments contributed to the combination treatment outcome, we then separated DCs and macrophages according to their location in the tumor or stromal compartment (Additional file 1: **Table S3**). We found that the high level of DCs located in the tumor compartment (defined as tumor DCs) at baseline, but not the DCs located in the stromal compartment (defined as stromal DCs), was associated with improved OS (*p* = 0.040, **Figure 1H**). High level of tumor macrophages at baseline had a tendency to be correlated with better OS (*p* = 0.054, **Figure 1I**). On the contrary, a high level of stomal DCs during the combination was related to poor PFS (*p* = 0.018, **Figure 1J**). These results demonstrated that the DCs and macrophages located in the tumor compartment played an important role in antitumor response in ESCC patients receiving combined chemoradiotherapy and PD-1 blockade.

### PD-L1^-^ DCs and Macrophages in the Tumor Compartment Associated With Improved Survival

Using multi-immunofluorescence assay, we could identify the PD-L1 expression on tumor cells, DCs, and macrophages simultaneously (4) (**Figures 1C–G**). Of the total PD-L1 expressed cells, the median percentages of tumor cells, DCs, and macrophages were 30.48%, 28.54%, and 15.44%, individually (Additional file 1: **Figure S1A**). PD-L1^+^ tumor cells decreased significantly (30.48% vs. 5.46%, *p* = 0.008) after the combination (Additional file 1: **Figures S1B, C**).

**Figure 2:**
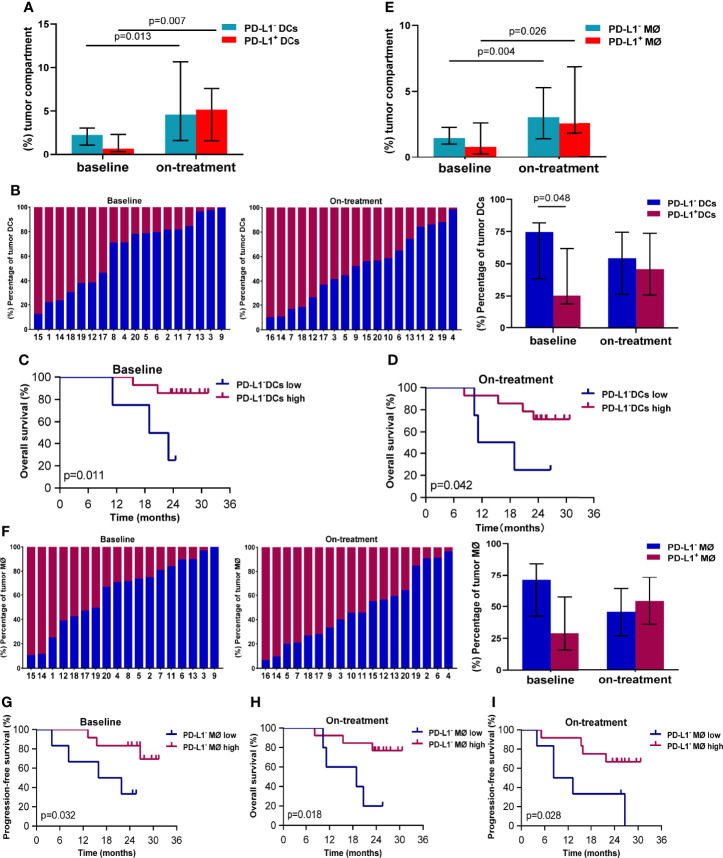
PD-L1^-^ dendritic cells and macrophages in the tumor compartment associated with better survival. **(A)** Proportion of PD-L1^-^ and PD-L1^+^ dendritic cells in the tumor compartment. **(B)** Ratio between PD-L1^-^ and PD-L1^+^ dendritic cells in the tumor compartment. **(C, D)** Kaplan–Meier curves showing overall survival based on PD-L1^-^ dendritic cells in the tumor compartment **(C)** at baseline **(D)** and during treatment. **(E)** Proportion of PD-L1^-^ and PD-L1^+^ macrophages in the tumor compartment. **(F)** Ratio between PD-L1^-^ and PD-L1^+^ macrophages in the tumor compartment. **(G, I)** Kaplan–Meier curves showing overall or progression-free survival based on PD-L1^-^ macrophages in the tumor compartment **(G)** at baseline and **(H, I)** during treatment. The tumors are ordered by the percentage of PD-L1^+^ dendritic cells or macrophages, from highest to lowest. Cutoff value: **(C)** ≥1.058%; **(D)** ≥1.469%; **(G)** 1.214%; **(H)** 1.713%; **(I)** 2.328%. On-treatment, after 40 Gy radiation. *p* ≤ 0.05, statistically significant.

Because of the close associations between the survival and APCs in the tumor compartment in our above finding, we here focused on PD-L1 expression on the APCs in the tumor compartment. The median percentage of DCs in the tumor compartment was 3.524% (95% CI, 2.754%–6.684%) at baseline, and increased to 11.394% (95% CI, 8.295%–23.439%) during combination treatment (Additional file 1: **Figure S2A**). The median proportions of PD-L1^-^ and PD-L1^+^ DCs in the tumor compartments were 2.297% (95% CI, 1.424%–3.927%) and 0.685% (95% CI, 0.425%–3.663%) at baseline (**Figure 2A**), and 4.579% (95% CI, 3.149%–16.728%) and 5.160% (95% CI, 3.322-8.896%) during the combination (**Figure 2A**), respectively. The PD-L1 expression on the tumor DCs showed great variability in individuals both at baseline and during the combination treatment (**Figure 2B**). The percentage of PD-L1^-^ DC was higher than that of PD-L1^+^ DCs in baseline tumor compartments (74.73% vs. 25.27%, *p* = 0.048, **Figure 2B**). While this difference disappeared during the combination treatment (**Figure 2B**). The Kaplan–Meier analysis showed that high levels of PD-L1^-^ DCs in both baseline and on-treatment tumor compartments were associated with improved OS (baseline, *p* = 0.011, **Figure 2C**; on-treatment, *p* = 0.042, **Figure 2D**). However, the PD-L1^+^ DCs in baseline or on-treatment tumor compartments were not related with survival (Additional file 1: **Figure S3**).

The median percentage of macrophages in the tumor compartment was 2.156% (95% CI, 1.786%–4.850%) at baseline, and increased to 5.822% (95% CI, 4.917%–11.415%) during combination treatment (Additional file 1: **Figure S2B**). The median proportions of PD-L1^-^ and PD-L1^+^ macrophages in tumor compartments were 1.464% (95% CI, 1.043%–2.669%) and 0.790% (95% CI, 0.469%–2.455%) at baseline (**Figure 2E**), which elevated to 3.043% (95% CI, 2.382%–5.290%) and 2.376% (95% CI, 1.694%–6.964%) during the combination (**Figure 2E**). Similar to DCs, tumor macrophages also exhibited heterogenous PD-L1 expression inter-individuals (**Figure 2F**). Patients with high level of PD-L1^-^ macrophages in baseline tumor compartments had longer PFS (*p* = 0.032, **Figure 2G**). Patients having high level of PD-L1^-^ macrophages in on-treatment tumor compartments had better OS (*p* = 0.018, **Figure 2H**) and PFS (*p* = 0.028, **Figure 2I**). These results suggested that the PD-L1^-^ DCs and macrophages in the tumor compartments promoted antitumor efficacy of chemoradiotherapy combined with PD-1 blockade in ESCC.

We also investigated the association between dendritic cells and macrophages in the tumor compartment. We found loose correlation between dendritic cells and macrophages in the on-treatment tumor compartment (Spearman coefficient 0.484, *p* = 0.042, Additional file 1: **Figure S4A**). The close relationship was observed between PD-L1^+^ dendritic cells and PD-L1^+^ macrophages in both baseline and on-treatment tumor compartment (Spearman coefficient 0.899 and 0.905, *p* < 0.001, Additional file 1: **Figures S4B, C**).

### Nearest Distance From Tumor Cells to PD-L1^-^ or PD-L1^+^ DCs and Macrophages

**Figure 3:**
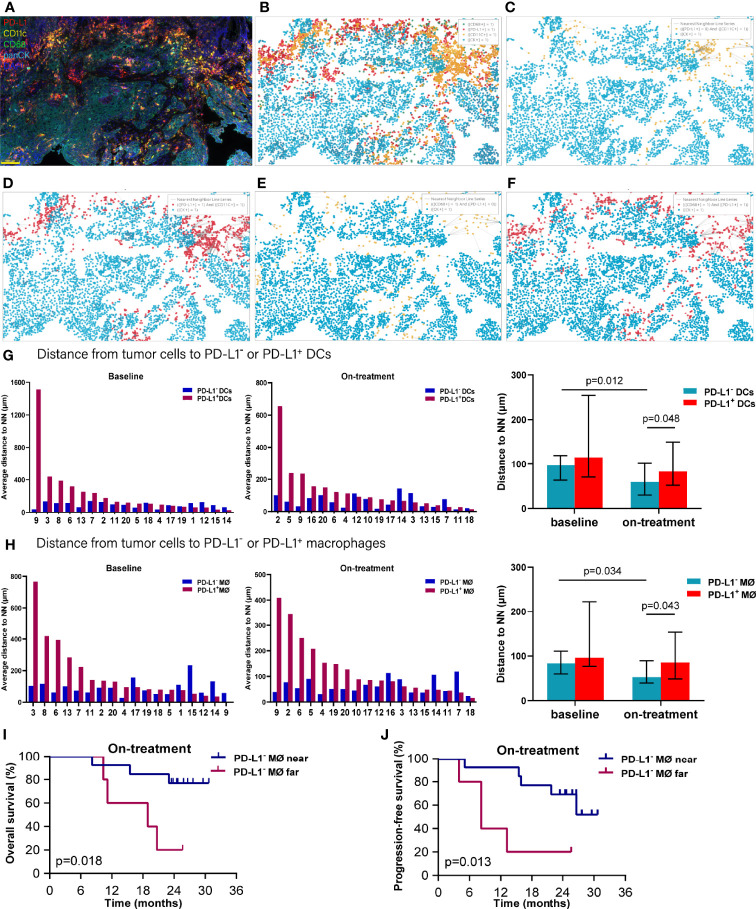
Distance from tumor cells to the nearest dendritic cells and macrophages. **(A)** Representative multiplex multi-immunofluorescence image (case N14, before treatment) showing staining for CD11 (yellow), CD68 (green), PD-L1 (red), and CK (cyan). **(B)** Cellular phenotype map of image shown in **A** depicting the locations of CK^+^ tumor cells (cyan dots), PD-L1^+^ (red dots), CD11c^+^ dendritic cells (orange dots), and CD68^+^ macrophages (green dots). **(C)** Ray plot depicting the distance from each CK^+^ tumor cell to the nearest PD-L^-^ dendritic cells. **(D)** Ray plot depicting the distance from each CK^+^ tumor cell to the nearest PD-L^+^ dendritic cells. **(E)** Ray plot depicting the distance from each CK^+^ tumor cell to the nearest PD-L^-^ macrophages. **(F)** Ray plot depicting the distance from each CK^+^ tumor cell to the nearest PD-L^+^ macrophages. **(G, H)** Distances from tumor cells to the nearest PD-L1^-^ and PD-L1^+^ dendritic cells **(G)** and macrophages **(H)** for all patients with available tumors at baseline and during treatment. **(I, J)** Kaplan–Meier curves showing overall survival **(I)** and progression-free survival **(J)** based on distance from tumor cells to the nearest PD-L1^-^ macrophages during treatment. The tumors are ordered by the percentage of PD-L1^+^ dendritic cells or macrophages, from highest to lowest. Cutoff: **(I)** 83.454 μm; **(J)** 83.454 μm. On-treatment, after 40 Gy radiation. *p* ≤ 0.05, statistically significant.

As both the compartment distribution and PD-L1^-^ or PD-1^+^ APCs inconsistently contributed to the outcome of the combination treatment, we next quantified the dynamical spatial relationship between tumor cells and these APC subpopulations, respectively. By using spatial multi-immunofluorescence analysis, we identified the coordinate position of the cells of each tissue section, and measured the distances from each tumor cell to the nearest PD-L1^-^ or PD-L1^+^ DCs or macrophages. The index of nearest distance was defined as the average of the closest distances from all tumor cells to the neighbors of each tissue section (**Figures 3A–F**). The distance from tumor cells to PD-L1^+^ DCs exhibited much more variability compared with that from tumor cells to PD-L1^-^ DCs (**Figure 3G**). Under the combination treatment, PD-L1^-^ DCs moved nearer to tumor cells compared with the corresponding ones at baseline (*p* = 0.012). The PD-L1^-^ DCs also located closer to tumor cells than the PD-L1^+^ DCs during the combination treatment (*p* = 0.048) (**Figure 3G** and **Table 1**). Similarly, a higher variability was found in the distance from tumor cells to the PD-L1^+^ macrophages (**Figure 3H**). The on-treatment PD-L1^-^ macrophages were closer to tumor cells than the baseline PD-L1^-^ macrophages and the on-treatment PD-L1^+^ macrophages (**Figure 3H** and **Table 1**). Kaplan–Meier analysis showed that patients with PD-L1^-^ macrophages nearer to tumor cells during the combination treatment had better OS (*p* = 0.018, **Figure 3I**) and PFS (*p* = 0.013, **Figure 3J**). These results showed that PD-L1^-^ DCs and macrophages locating adjacently to the tumor cells provided them spatial advantage to participate in the antitumor immune response under the combination treatment.

**Table 1:** Nearest distance from tumor cells to neighbors.

Neighbors	Distance at baseline (μm)	Distance after 40 Gy (μm)
PD-L1^-^ dendritic cell	91.08 (74.32–109.92)	50.40 (39.83–83.61)
PD-L1^+^ dendritic cell	114.93 (45.57–427.35)	73.70 (44.32–210.93)
PD-L1^-^ macrophage	74.48 (62.80–116.38)	52.72 (46.26–76.35)
PD-L1^+^ macrophage	95.91 (64.19–266.03)	85.27 (71.08–194.41)

Data are median (95% CI). HALO^®^ image analysis platform (Indica Labs, USA) was used in spatial analysis.

### Nearest Distance From PD-L1^-^ and PD-L1^+^ Tumor Cells to DCs and Macrophages

**Figure 4:**
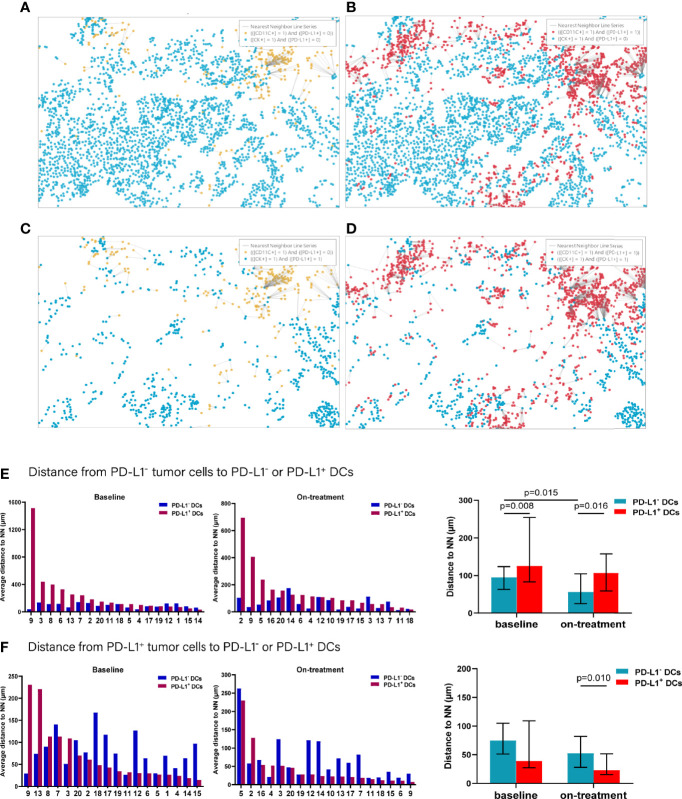
Distance from PD-L1^-^ or PD-L1^+^ tumor cells to the nearest PD-L1^-^ or PD-L1^+^ dendritic cells. **(A–D)** Spatial analysis shown in **Figure 3A**. **(A)** Ray plot depicting the distance from each CK^+^PD-L1^-^ tumor cell to the nearest PD-L^-^ dendritic cell. **(B)** Ray plot depicting the distance from each CK^+^PD-L1^-^ tumor cell to the nearest PD-L1^-^ dendritic cells. **(C)** Ray plot depicting the distance from each CK^+^PD-L1^+^ tumor cell to the nearest PD-L^-^ dendritic cell. **(D)** Ray plot depicting the distance from each CK^+^PD-L1^+^ tumor cell to the nearest PD-L^+^ dendritic cells. **(E)** Distances from PD-L1^-^ tumor cells to the nearest PD-L1^-^ or PD-L1^+^ dendritic cells at baseline and during treatment. **(F)** Distances from PD-L1^+^ tumor cells to the nearest PD-L1^-^ or PD-L1^+^ dendritic cells at baseline and during treatment. The tumors are ordered by the percentage of PD-L1^+^ dendritic cells, from highest to lowest. On-treatment, after 40 Gy radiation. *p* ≤ 0.05, statistically significant.

To further explore the interaction between tumor cells and the DCs and macrophages, we next assessed their spatial relationship by dividing the tumor cells and APCs into PD-L1^-^ and PD-L1^+^ subpopulations, respectively (**Figures 4A–D**). Firstly, we calculated the distance from each PD-L1^-^ tumor cells to the nearest PD-L1^-^ and PD-L1^+^ DCs. We found that the PD-L1^-^ DCs located closer to the PD-L1^-^ tumor cells compared with PD-L1^+^ DCs both at baseline and during the combination treatment (baseline, *p* = 0.008; on-treatment, *p* = 0.016, **Figure 4E** and **Table 2**). After the combination treatment, the PD-L1^-^ DCs further moved nearer to the PD-L1^-^ tumor cells than those at baseline (*p* = 0.015, **Figure 4E** and **Table 2**). Next, we analyzed the distance from PD-L1^+^ tumor cells to PD-L1^-^ and PD-L1^+^ DCs. The distance from the PD-L1^+^ tumor cells to PD-L1^-^ or PD-L1^+^ DCs did not change significantly under the combination treatment. However, the PD-L1^+^ DCs located closer to PD-L1^+^ tumor cells than PD-L1^-^ DCs during the combination treatment (*p* = 0.010, **Figure 4F** and **Table 2**).

**Table 2:** Nearest distance from PD-L1^-^ and PD-L1^+^ tumor cells to neighbors.

Neighbors	Distance at baseline (μm)		Distance after 40 Gy (μm)	
	PD-L1^-^ tumor	PD-L1^+^ tumor	PD-L1^-^ tumor	PD-L1^+^ tumor
PD-L1^-^ dendritic cell	84.29	74.73	45.53	52.53
	(72.94–108.60)	(58.08–103.02)	(37.40–87.29)	(36.01–103.68)
PD-L1^+^ dendritic cell	125.01	38.90	97.48	22.94
	(54.83–433.84)	(33.31–105.36)	(58.02–243.14)	(13.57–74.86)
PD-L1^-^ macrophage	72.10	83.58	53.53	53.29
	(59.45–111.65)	(63.30–136.44)	(44.46–70.63)	(47.44–95.56)
PD-L1^+^ macrophage	24.51	40.09	66.81	29.70
	(18.18–64.10)	(25.08–106.47)	(14.71–357.54)	(25.42–78.25)

Data are median (95% CI). HALO^®^ image analysis platform (Indica Labs, USA) was used in spatial analysis.

**Figure 5:**
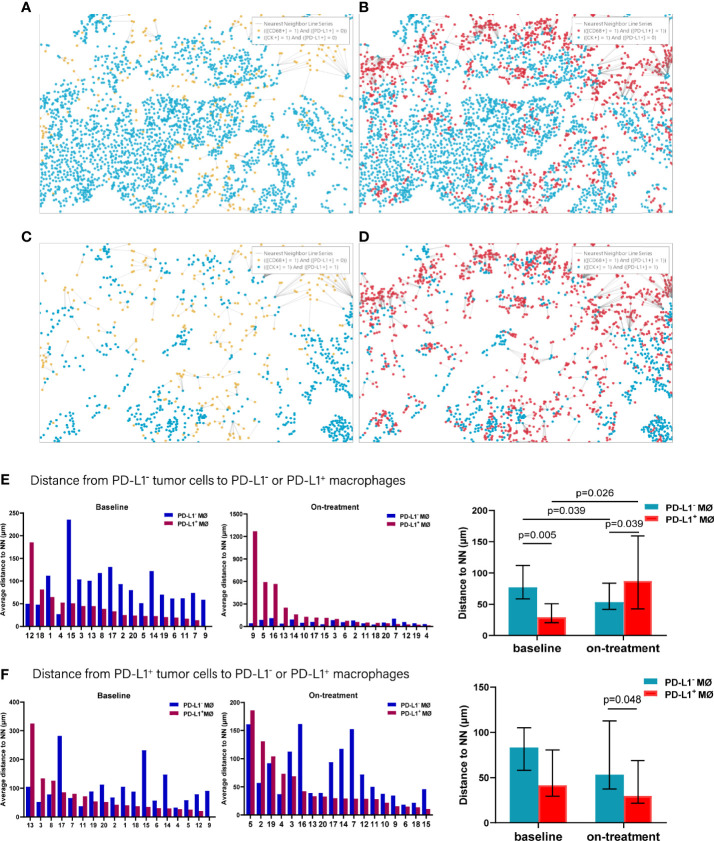
Distance from PD-L1^-^ or PD-L1^+^ tumor cells to the nearest PD-L1^-^ or PD-L1^+^ macrophages. **(A–D)** Spatial analysis shown in **Figure 3**. **(A)** Ray plot depicting the distance from each CK^+^PD-L1^-^ tumor cell to the nearest PD-L1^-^ macrophages. **(B)** Ray plot depicting the distance from each CK^+^PD-L1^-^ tumor cell to the nearest PD-L1^+^ macrophages. **(C)** Ray plot depicting the distance from each CK^+^PD-L1^+^ tumor cell to the nearest PD-L^-^ macrophages. **(D)** Ray plot depicting the distance from each CK^+^PD-L1^+^ tumor cell to the nearest PD-L1^+^ macrophages. **(E)** Distances from PD-L1^-^ tumor cells to the nearest PD-L1^-^ or PD-L1^+^ macrophages at baseline and during treatment. **(F)** Distances from PD-L1^+^ tumor cells to the nearest PD-L1^-^ or PD-L1^+^ macrophages at baseline and during treatment. The tumors are ordered by the percentage of PD-L1^+^ macrophages, from highest to lowest. On-treatment, after 40 Gy radiation. *p* ≤ 0.05, statistically significant.

In analyzing the distance from PD-L1^-^ tumor cells to PD-L1^-^ and PD-L1^+^ macrophages (**Figures 5A–D**), we found that the PD-L1^-^ macrophages were farther away from the PD-L1^-^ tumor cells compared with PD-L1^+^ macrophages at baseline (*p* = 0.005, **Figure 5E** and **Table 2**). PD-L1^-^ macrophages migrated closer to PD-L1^-^ tumor cells (*p* = 0.039), while PD-L1^+^ macrophages moved away from PD-L1^-^ tumor cells (*p* = 0.026) during the combination treatment (**Figure 5E** and **Table 2**). Consequently, opposite to the distances at baseline, PD-L1^-^ macrophages got closer to PD-L1^-^ tumor cells than PD-L1^+^ macrophages during combination treatment (*p* = 0.039, **Figure 5E** and **Table 2**). We then analyzed the distance from PD-L1^+^ tumor cells to PD-L1^-^ and PD-L1^+^ macrophages. The distance from PD-L1^+^ tumor cells to PD-L1^-^ macrophages as well as to PD-L1^+^ macrophages did not alter under the combination treatment (**Figure 5F** and **Table 2**). However, PD-L1^+^ macrophages located nearer to PD-L1^+^ tumor cells than PD-L1^-^ macrophages during the combination treatment (*p* = 0.048, **Figure 5F**, and **Table 2**).

**Figure 6:**
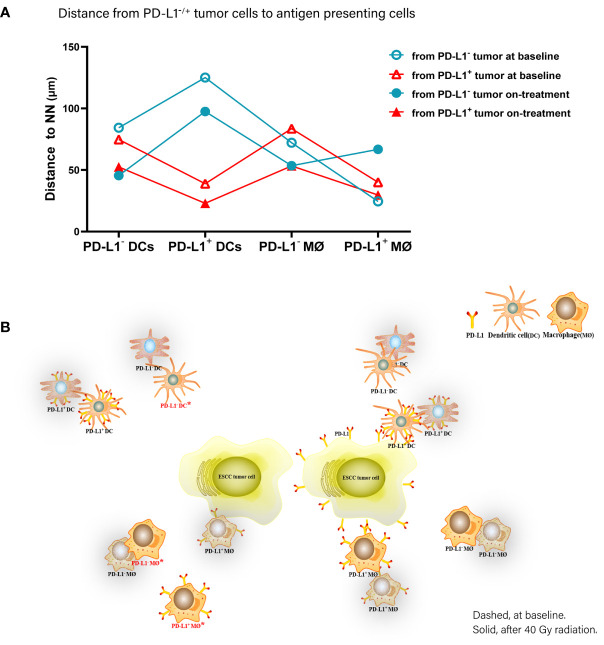
Spatial distribution pattern dendritic cells and macrophages in ESCC patients under chemoradiotherapy combined with PD-1 blockade. **(A)** Dynamic alteration of distance from PD-L1^-^ or PD-L1^+^ tumor cells to the nearest PD-L1^-^ or PD-L1^+^ dendritic cells and macrophages in ESCC patients under combination treatment. **(B)** Model of dynamic spatial distribution of dendritic cells and macrophages in ESCC under treatment. Dashed, at baseline. Solid, after 40 Gy radiation. ^*^, statistical significance of baseline distance from tumor cells to dendritic cells and macrophages compared with on-treatment.

Accordingly, of the APC subsets located relative to PD-L1^-^ tumor cells, PD-L1^+^ macrophages were the nearest at baseline, while PD-L1^-^ DCs and macrophages turned to the nearest during the combination treatment. Inconsistently, PD-L1^+^ DCs and macrophages were the nearest APC subsets to PD-L1^+^ tumor cells both before and under the combination treatment (**Figure 6**). These results further proved the distinct spatial pattern of APC subpopulations in ESCC, which was also modulated by chemoradiotherapy combined with PD-1 blockade.

### Predictive Significance of Spatial Distribution of DC and Macrophage Subsets

**Figure 7:**
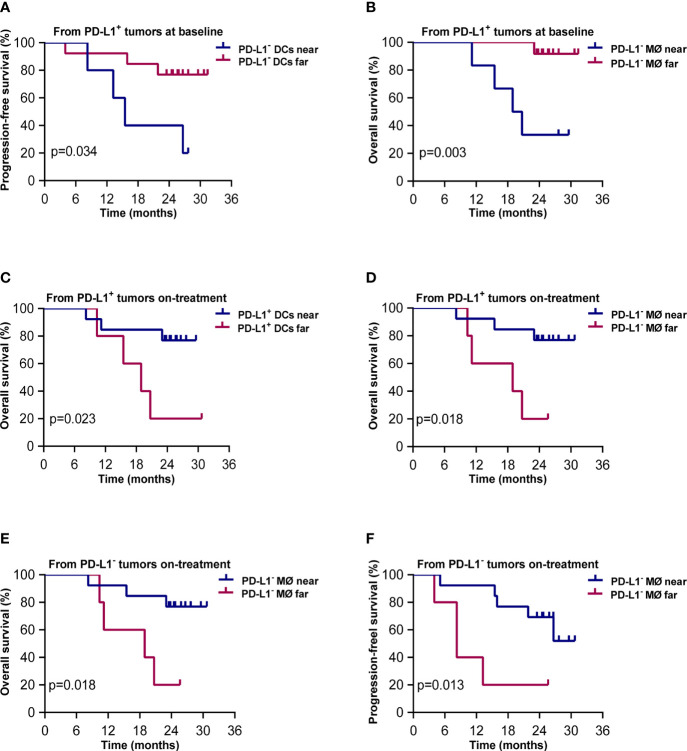
Spatial distribution of dendritic cell and macrophage subsets associated with survival. Kaplan–Meier curves showing overall and progression-free survival based on distance from **(A)** PD-L1^+^ tumor cells to the nearest PD-L1^-^ dendritic cells at baseline; **(B)** PD-L1^+^ tumor cells to PD-L1^-^ macrophages at baseline; **(C)** PD-L1^+^ tumor cells to the nearest PD-L1^+^ dendritic cells during treatment; **(D)** PD-L1^+^ tumor cells to the nearest PD-L1^-^ macrophages during treatment; **(E)** PD-L1^-^ tumor cells to the nearest PD-L1^-^ macrophages during treatment; **(F)** PD-L1^-^ tumor cells to the nearest PD-L1^-^ macrophages during treatment. Cutoff value: **(A)** 57.694 μm; **(B)** 66.762 μm; **(C)** 49.136 μm; **(D)** 103.159 μm; **(E)** 81.396 μm; **(F)** 81.396 μm. On-treatment, after 40 Gy radiation. *p* ≤ 0.05, statistically significant.

The results of Kaplan–Meier analysis showed that shorter distances from PD-L1^+^ tumor cells to PD-L1^-^ DCs and to PD-L1^-^ macrophages at baseline were associated with worse PFS and OS, respectively (*p* = 0.034, *p* = 0.003, **Figures 7A, B**). On the contrary, shorter distances from PD-L1^+^ tumor cells to PD-L1^+^ DCs and PD-L1^-^ macrophages during the combination treatment were both correlated with better OS (*p* = 0.023, *p* = 0.018, **Figures 7C, D**). Shorter distances from PD-L1^-^ tumor cells to PD-L1^-^ macrophages during the combination treatment also predicted improved OS (*p* = 0.018, **Figure 7E**) and PFS (*p* = 0.008, **Figure 7F**). These results elicited that the close interaction between tumor cells and APCs with different PD-L1 expression contributed to divergent outcome of chemoradiotherapy combined with PD-1 blockade in ESCC.

### Spatial Distribution of DCs and Macrophages Associated With Tumor Mutation Burden

**Figure 8:**
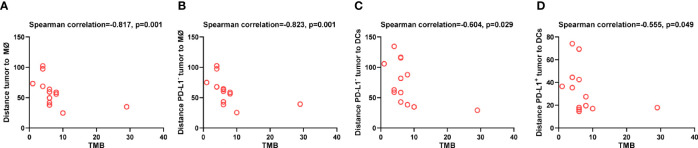
Spatial distribution of dendritic cells and macrophages associated with tumor mutation burden. Spearman correlation analysis between tumor mutation burden and distance from **(A)** tumor cells to the nearest macrophages; **(B)** PD-L1^-^ tumor cells to the nearest macrophages; **(C)** PD-L1^-^ tumor cells to the nearest dendritic cells; **(D)** PD-L1^+^ tumor to the nearest dendritic cells. *p* ≤ 0.05, statistically significant.

Lastly, to explore the tumor-derived factors that might affect the distribution of DCs and macrophages, we evaluated the association between TMB and the distance of these APCs to tumor cells. We found that higher TMB was associated with shorter distance between macrophages and tumor cells (*p* = 0.001, **Figure 8A**), especially between macrophages and PD-L1^-^ tumor cells (*p* = 0.001, **Figure 8B**). Similarly, higher TMB was correlated with shorter distance between DCs and PD-L1^-^ tumor cells (*p* = 0.029, **Figure 8C**), as well as between DCs and PD-L1^+^ tumor cells (*p* = 0.049, **Figure 8D**). The association between high TMB and far distance of the APCs to tumor cells was not observed. These results indicated that high TMB would result in closer distribution of APCs to tumor cells.

## Discussion

This is the first study to dynamically illustrate the spatial pattern of DCs and macrophages in ESCC patients treated with combined chemoradiotherapy and immunotherapy. The results showed a detailed description of the distinct spatial distribution of DCs and macrophages in ESCC. Chemoradiotherapy combined with PD-1 blockade promoted these APCs to migrate closer to tumor cells. The close distance between APCs and tumor cells during the combination predicted improved outcome.

We found that the DCs and macrophages in the baseline tumor compartment, but not the stromal compartment were associated with better survival. The heterogeneity of tumor and stromal profiling reflected the divergent response to immunotherapy (21). The antitumor function of DCs and macrophages was usually dysregulated by various factors derived from both the tumor cells and the immune inhibitory tumor microenvironment (22, 23). However, chemoradiotherapy could remodel the inflammatory tumor microenvironment (15), where APCs recovered their capacity in the antitumor immune response. The higher APCs in the baseline tumor compartment in our findings indicated higher antitumor potential in these ESCC patients under chemoradiotherapy combined with PD-1 blocked. These results also suggested that these APCs should arrive near enough to tumor cells to phagocytose and present tumor neoantigens, thus triggering an antitumor immune response.

Consistently, our spatial analysis showed the close distribution of DCs and macrophages around tumor cells during the combination predicted longer survival. However, comparing the survival analysis in APC percentages in tumor tissues, the spatial analysis illustrated more detailed mechanisms of APC-primed antitumor immune response induced by combination. The basic step of APCs generates the adaptive immune response that is effective for antigen acquisition and processing (24). The close distribution of APCs around tumor cells promoted APCs that effectively uptake the tumor antigens as well as sense alarming factors from the dying tumor cells under chemoradiotherapy (25). Our combination strategy blocked the PD-1 signaling during the activation of T cells upon TCR recognition of peptide/major histocompatibility complex class II complex displayed on APCs, probably synergizing the antitumor effect. Although radiotherapy evoked antitumor immune response, the proliferated T, B, and NK cells activated during radiotherapy were sensitive to radiation-induced cytotoxicity. Additionally, the on-treatment tumor biopsies were always collected during radiotherapy (5, 26). As a result, poor relationship between the on-treatment tumor-infiltrating T cells and patient survival was observed in our present and previous study (4) (26). Identifying the functional status might provide clues of the antitumor immune characteristics of these radiosensitive immune cells in further studies. On the contrary, DCs and macrophages were more resistant to radiation compared with T, B, and NK cells (27), which could more accurately reflect the immune status under radiotherapy. Our results demonstrated that close distance between DCs and macrophages and irradiated tumor cells benefit these APCs in presenting more released neoantigens, and promoting antitumor immune response. These results provided new evidence that spatial measurement of tumor-infiltrated APCs during treatment could be a potential predictive biomarker in immunotherapy combined with the conventional therapeutic strategies.

We found that the negative PD-L1 on DCs and macrophages was critical to improve combination outcome. Multiple cytokines in the tumor microenvironment, such as the type I and II interferon, IL-6, and CXCL8, could elevate PD-L1 expression on DCs and macrophages (28–30). PD-L1 on DCs and macrophages played an important role in limiting T-cell response and promoting immune evasion (12, 29, 31). Anti-PD-1 antibody blocked PD-L1/PD-1 interaction, thus facilitating re-activation of the tumor-infiltrated T cells for tumor control. However, despite successes in the clinic, most patients do not respond to PD-1 blockade. Recent studies revealed the underlying mechanisms beyond APC-PD-L1 binding T cell-PD-1 in trans, which resulted in the ineffective response under PD-1 blockade (32–34). Besides expressing on T cells, PD-1 is co-expressed with PD-L1 on APCs. The co-expressed PD-1 binds to PD-L1 in cis attenuated PD-L1 signaling in T cells. If anti-PD-1 antibody unselectively blockaded PD-1 on both T cells and APCs, the PD-L1 on APCs would be free to inhibit T-cell signaling and cytotoxicity (32). Meanwhile, another PD-L1 ligand CD80 (B7.1) was widely expressed on DCs and macrophages (33, 35). The cis-PD-L1/CD80 binding on DCs sequestrated CD80 interaction with CD28 to enhance T-cell priming (33). PD-L1 expression also restrained DC maturation and macrophage M1 polarization (34). Several studies have demonstrated that PD-L1-expressing APCs rather than tumor cells played an essential role in anti-PD-L1 monotherapy in preclinical tumor models (36, 37). In our study, although PD-1 blockade was concurrently used with chemoradiotherapy, negative PD-L1 on APCs was vital to better survival. It was probably that the abundant PD-L1 on APCs not only inhibited activation of T cells by PD-L1/PD-1 interaction, but also damped T-cell priming by PD-L1/CD80 binding under PD-1 blockade. These results noted that PD-L1 expression and functional status of APCs need to be included in exploration of biomarkers in spatial analysis. For patients who were resistant to chemoradiotherapy combined with PD-1 blockade and had high PD-L1 expression on APCs, adding PD-L1 inhibitor might reverse the treatment resistance and improve outcome. Additionally, our results also indicated that close distribution of PD-L1^+^ DCs to PD-L1^+^ tumor cells during the combination treatment could benefit the patient survival. It was probably that gradient distribution of antitumor cytokines, such as interferon-γ, upregulated PD-L1 expression on both the tumor and DCs.

In dynamically monitoring the spatial distribution of DCs and macrophages, we observed tumor tropism of these APCs during the combination treatment, although the distance changes between the APCs and PD-L1^+^ tumor cells did not reach the significant difference probably because of limited patients included. Radiation led to tumor immunogenic cell death, increased the release of damage-associated molecular patterns, and consequently activated adaptive immune response (38–40). Radiation promoted the release of tumor antigens displayed on a tumor cell surface and elevated antigen expression to levels sufficient for cross-presentation, thus increasing the number of DCs presenting antigens (41, 42). Meanwhile, radiation activated inflammatory pathways (43). Our results indicated that the remodeling of the tumor microenvironment by chemoradiotherapy combined with immunotherapy attracted more APCs to infiltrate into the irradiated tumor site, which provided space superiority for these cells effectively triggering antitumor response. Interestingly, we found that PD-L1^-^ DCs and macrophages preferred to surround PD-L^-^ tumor cells, while PD-L1^+^ DCs and macrophages tended to locate around PD-L^+^ tumor cells. The intratumor heterogeneity in ESCC, including genomic mutation and epigenomic aberrations (44–46), contributed to the heterogenetic immune characteristics under PD-1 blockade combined with chemoradiotherapy. How these heterogenetic response affected the combination outcome and the multi-regional communication deserves further study.

Finally, we found that a high TMB was associated with the close distribution of the APCs to tumor cells at baseline. High TMB tumors had the high possibility to produce more tumor neoantigens, thus elevating antigen presentation and inducing an antitumor immune response (47). TMB might alter under radiotherapy (48). The TMB that was assessed in the baseline tumor was probably inconsistent with what it was during the treatment. This might partially explain the result that we did not find an association between TMB and APC distribution during the combination treatment. Nevertheless, combining the finding of closer distribution of APCs around tumor cells during combination, we highlighted that tumor tropism of APCs promoted by increased release of tumor neoantigens was one of the most important antitumor mechanisms in this combination strategy in ESCC.

Nevertheless, this study also had several limitations. Firstly, since the biopsies were collected from the phase Ib study, the number of biopsies was limited. The matched baseline and on-treatment biopsies collected in this study could in part decrease the bias. Secondly, the M1 and M2 macrophages were not distinguished in the study. Because M1 and M2 macrophage polarization was flexibly regulated by the stimuli in inflammatory environment (49), we applied the functional marker PD-L1 rather than phenotype markers of M1 and M2 macrophages in the present study. Thirdly, although low overlap between CD11c and CD68 was observed, it needs to be considered in further studies (Additional file 1: **Figure S5**). The roles of multiple subpopulations of DCs and macrophages in tumor microenvironment are worth investigating.

Conclusively, our findings reveal that close spatial distribution between tumor cells and DCs and macrophages is critical in the combination efficiency of chemoradiotherapy and PD-1 blockade in ESCC. The spatial distribution patterns of tumor-infiltrating APCs are biomarker candidates in this combination treatment in ESCC, and the underlying mechanisms need to be further studied.

## Data Availability Statement

The original contributions presented in the study are included in the article/**Supplementary Material**. Further inquiries can be directed to the corresponding authors.

## Ethics Statement

The studies involving human participants were reviewed and approved by the Institutional Review Board and Ethics Committee at Tianjin Medical University Cancer Institute and Hospital. The patients/participants provided their written informed consent to participate in this study.

## Author Contributions

Study concept and design: WZ and CY. Experiments: XM, ZG, XW, DH, TZ, XC, FC, and JD. Data analysis and interpretation of data: WZ, CY, XM, GZ, LZ, ZY, PW, and QP. Drafting the manuscript: WZ, CY, and XM. Critical review of the manuscript: WZ and CY. All authors contributed to the article and approved the submitted version.

## Funding

This work was supported by the Chinese National Key Research and Development Project (No. 2018YFC1315601) and the National Nature Science Foundation of China (grants 81872462, 81972772, and 82073348).

## Conflict of Interest

The authors declare that the research was conducted in the absence of any commercial or financial relationships that could be construed as a potential conflict of interest.

## Publisher’s Note

All claims expressed in this article are solely those of the authors and do not necessarily represent those of their affiliated organizations, or those of the publisher, the editors and the reviewers. Any product that may be evaluated in this article, or claim that may be made by its manufacturer, is not guaranteed or endorsed by the publisher.
