# Supplementary material for: Spatial Distribution and Predictive Significance of Dendritic Cells and Macrophages in Esophageal Cancer Treated With Combined Chemoradiotherapy and PD-1 Blockade
Source: Front Immunol. 2022 Jan 3;12:786429. doi: 10.3389/fimmu.2021.786429 (PMC8761740; doi:10.3389/fimmu.2021.786429)
Supplement: Supplementary file 1 [file DataSheet_1.docx]

**Supplementary file 1**

**Table S1. Samples available for exploration analysis**

| **Patient ID** | **FoundationOne CDx (Before the treatment)** | **Multiplex IF (Before the treatment)** | **Multiplex IF (During the treatment^a^)** |
| --- | --- | --- | --- |
| 1 | × | √ | × |
| 2 | √ | √ | √ |
| 3 | √ | √ | √ |
| 4 | √ | √ | √ |
| 5 | √ | √ | √ |
| 6 | √ | √ | √ |
| 7 | √ | √ | √ |
| 8 | × | √ | × |
| 9 | √ | √ | √ |
| 10 | × | ×^b^ | √ |
| 11 | √ | √ | √ |
| 12 | × | √ | √ |
| 13 | √ | √ | √ |
| 14 | √ | √ | √ |
| 15 | √ | √ | √ |
| 16 | √ | ×^b^ | √ |
| 17 | √ | √ | √ |
| 18 | √ | √ | √ |
| 19 | × | √ | √ |
| 20 | × | √ | √ |

^a^ after 40 Gy radiation. ^b^stained tumor section without tumor cells included. IF=immunofluorescence.

**Table S2: Baseline patient characteristics and treatment completion**

| **Characteristic** | **Data (n=20)** |
| --- | --- |
| Age, median (range), y | 64 (56–74) |
| Sex |  |
| Male | 18 (90) |
| Female | 2 (10) |
| ECOG performance status score |  |
| 0 | 16 (80) |
| 1 | 4 (20) |
| Smoking status |  |
| Never | 7 (35) |
| Former or current | 13 (65) |
| Drinking status |  |
| Never | 6 (30) |
| Former or current | 14 (56) |
| Location |  |
| Cervical segment | 2 (10) |
| Upper thoracic segment | 7 (35) |
| Middle thoracic segment | 8 (40) |
| Inferior thoracic segment | 3 (15) |
| AJCC8^a^ disease stage |  |
| I | 1 (5) |
| III | 6 (30) |
| IV | 13 (65) |
| AJCC6^b^ disease stage |  |
| IIb | 2 (10) |
| III | 13 (65) |
| IVa | 5 (25) |
| Treatment completion |  |
| Radiation = 60 Gy | 14 (70) |
| Radiation≥54 Gy | 20 (100) |
| Camrelizumab （PD-1 blockade） | 17 (85) |
| Cisplatin plus docetaxel≥ 3 cycles | 20 (100) |
| Apatinib ？？cycles | 11 (55) |

Data are n (%), unless otherwise specified. ^a^8^th^ (2017) version of the AJCC Staging Manual. ^b^6^th^ (2002) version of the AJCC Staging Manual. AJCC= American Joint Committee on Cancer. ECOG=Eastern Cooperative Oncology Group.

**Table S3. Number of immune cells counted in each tumor tissue by using multiplex immunofluorescence staining.**

| **Patient ID** | **Tumor compartment at baseline** | | | **Stromal compartment at baseline** | | | **Tumor compartment after 40 Gy radiation** | | | **Stromal compartment after 40 Gy radiation** | | |
| --- | --- | --- | --- | --- | --- | --- | --- | --- | --- | --- | --- | --- |
|  | **CK+** | **CD11c+** | **CD68+** | **CK+** | **CD11c+** | **CD68+** | **CK+** | **CD11c+** | **CD68+** | **CK+** | **CD11c+** | **CD68+** |
| 1 | 3540 | 906 | 450 | 70 | 760 | 281 | NA | NA | NA | NA | NA | NA |
| 2 | 25428 | 1002 | 1148 | 4323 | 1970 | 1630 | 972 | 173 | 250 | 0 | 1130 | 846 |
| 3 | 21216 | 771 | 466 | 280 | 1714 | 958 | 4185 | 267 | 295 | 487 | 5433 | 1583 |
| 4 | 7262 | 1803 | 2900 | 2156 | 6309 | 4950 | 17619 | 7738 | 2319 | 1991 | 2262 | 565 |
| 5 | 20394 | 820 | 643 | 4071 | 3781 | 1234 | 126 | 250 | 145 | 0 | 545 | 694 |
| 6 | 26556 | 733 | 1175 | 784 | 819 | 373 | 133 | 20 | 23 | 15 | 214 | 78 |
| 7 | 19731 | 208 | 641 | 1007 | 1226 | 469 | 28332 | 1704 | 1850 | 16543 | 15810 | 2859 |
| 8 | 12977 | 512 | 726 | 201 | 844 | 281 | NA | NA | NA | NA | NA | NA |
| 9 | 19379 | 2800 | 481 | 290 | 2258 | 310 | 23 | 44 | 12 | 5 | 11380 | 3592 |
| 10 | -^a^ | -^a^ | -^a^ | -^a^ | -^a^ | -^a^ | 1185 | 867 | 552 | 126 | 10470 | 5505 |
| 11 | 5101 | 443 | 196 | 226 | 461 | 97 | 4170 | 8034 | 785 | 2504 | 7654 | 1281 |
| 12 | 5548 | 1119 | 2026 | 1413 | 8032 | 8315 | 5590 | 399 | 781 | 632 | 667 | 559 |
| 13 | 3670 | 719 | 208 | 83 | 649 | 351 | 166 | 82 | 59 | 62 | 522 | 432 |
| 14 | 23075 | 4768 | 1630 | 1977 | 3804 | 1052 | 506 | 927 | 398 | 77 | 2540 | 1628 |
| 15 | 11378 | 1176 | 261 | 3914 | 3604 | 696 | 2051 | 2142 | 2599 | 2685 | 14423 | 10832 |
| 16 | -^a^ | -^a^ | -^a^ | -^a^ | -^a^ | -^a^ | 252 | 188 | 179 | 1 | 2993 | 1139 |
| 17 | 38219 | 4594 | 1814 | 561 | 878 | 391 | 3580 | 3446 | 2386 | 555 | 4439 | 2257 |
| 18 | 15324 | 832 | 1384 | 5 | 879 | 906 | 305 | 539 | 737 | 49 | 8964 | 8065 |
| 19 | 18136 | 1001 | 626 | 1458 | 1837 | 328 | 2334 | 2714 | 369 | 965 | 4549 | 592 |
| 20 | 12485 | 853 | 625 | 145 | 305 | 130 | 10581 | 913 | 1859 | 365 | 812 | 547 |

-^a^, stained tumor section without tumor cells included.

NA, no biopsy samples collected.


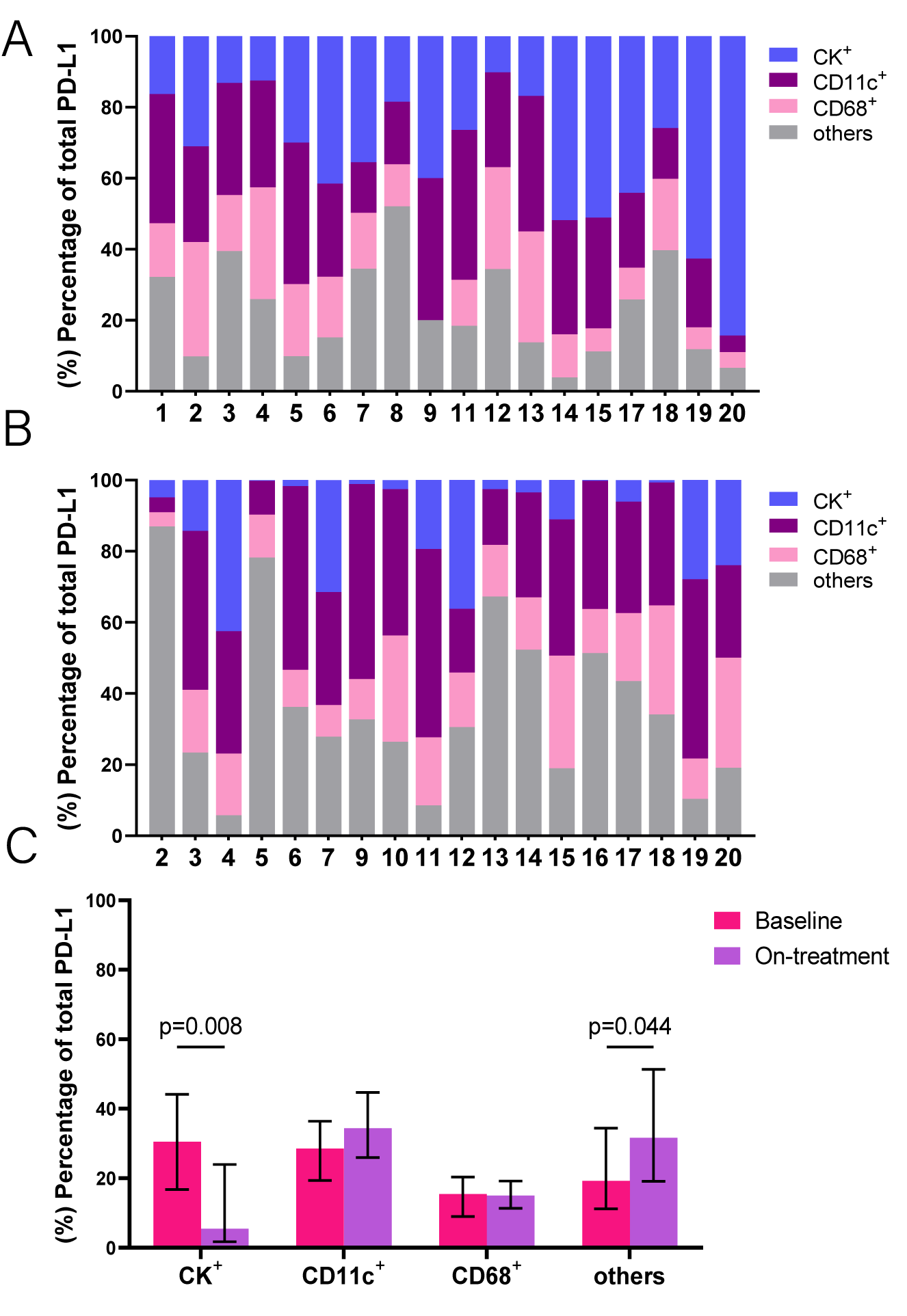


**Figure S1. Composition of PD-L1 expressed cells in tumor tissues.**

(A) Baseline. (B) During combination treatment (After 40 Gy radiation). (C) Statistic analysis using Wilcoxon Signed-Rank tests. *P* ≤ 0.05, statistically significant.


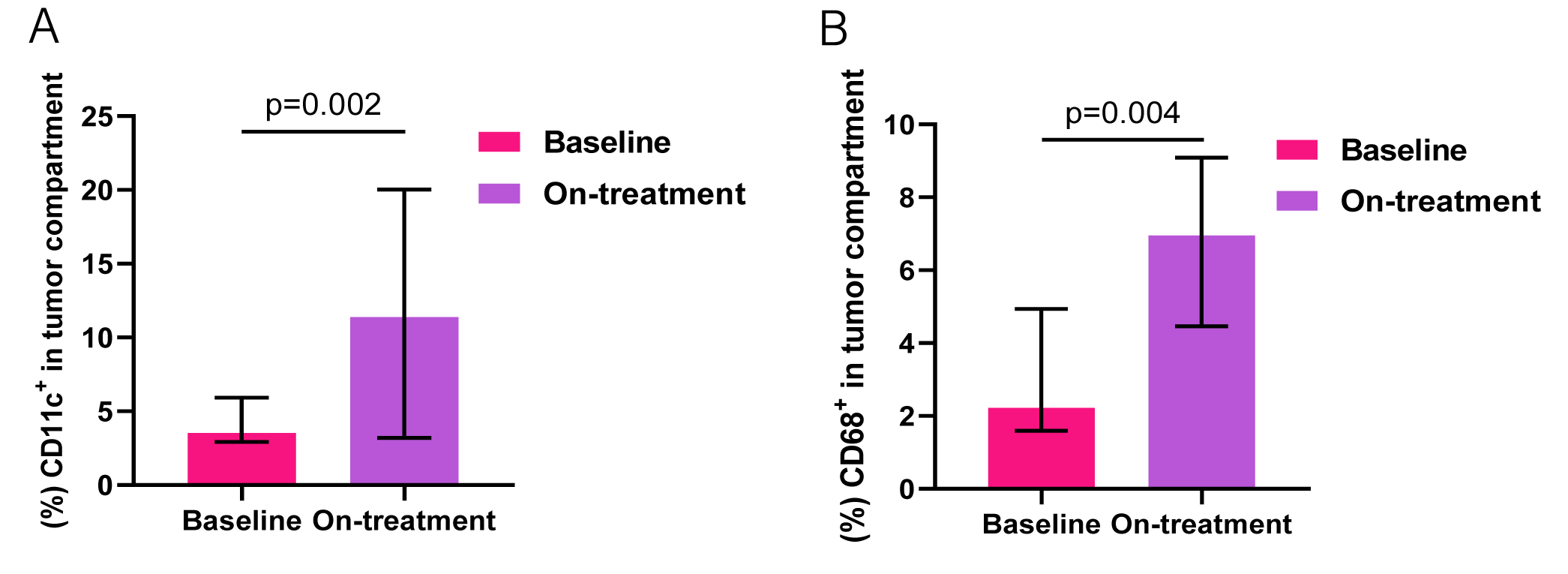


**Figure S2. Percentage of dendritic cells and macrophages in both baseline and on-treatment tumor compartments.**


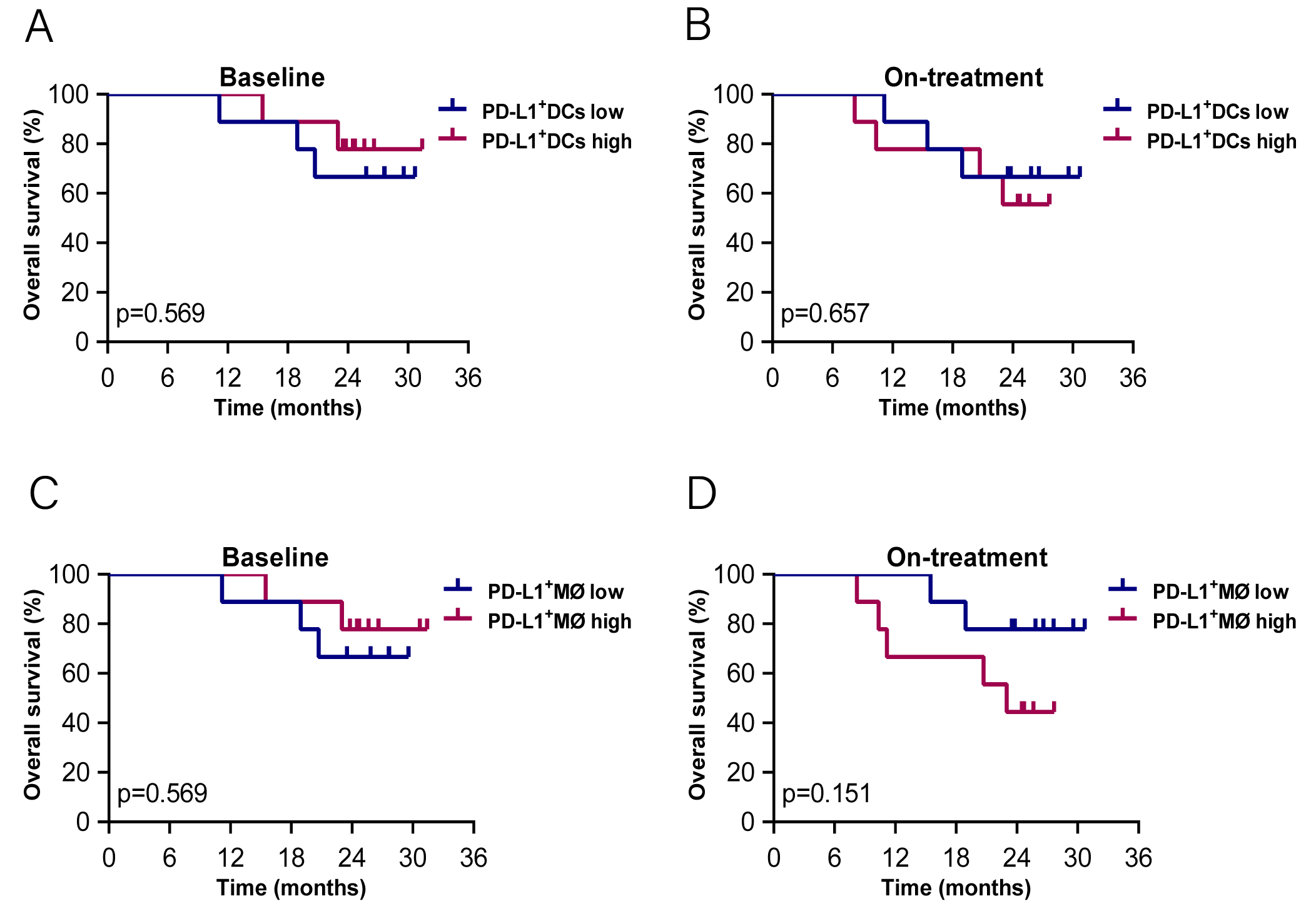


**Figure S3. Kaplan–Meier survival analysis in PD-L1+ dendritic cells and macrophages in the tumor compartments.**

Patient overall survival in PD-L1^+^ dendritic cells in baseline (A) and on-treatment (B) tumor compartments; PD-L1^+^ macrophages in baseline (C) and on-treatment (D) tumor compartments. All the best cutoff of Kaplan–Meier survival analysis was calculated by the Youden index of the ROC curve (D). If the patient numbers of the two groups were not balanced (more than the ratio of 10 to 3), median value was used as the cutoff value in Kaplan-Meier survival analysis (A ,B and C). P < 0.05, statistically significant.


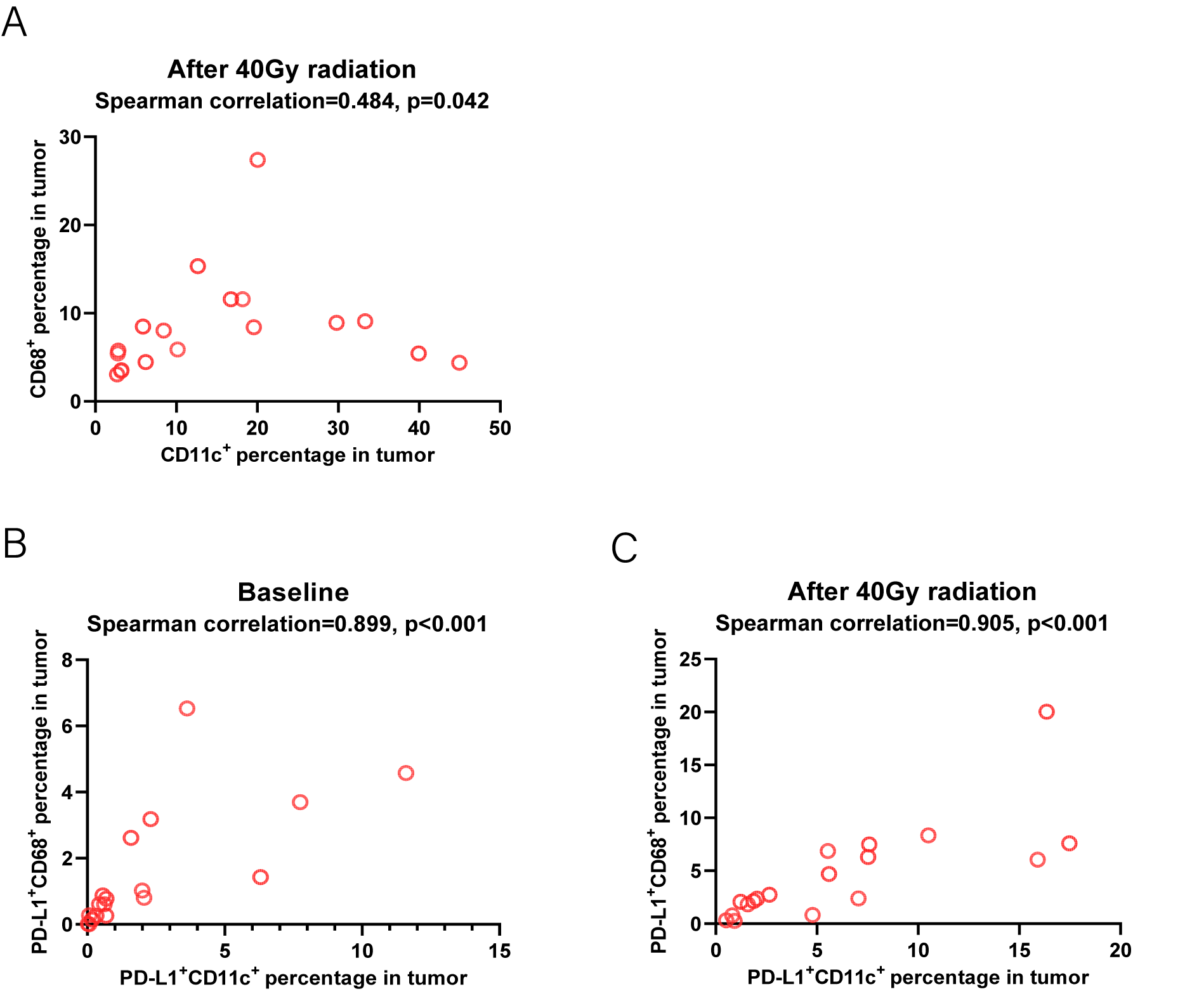


**Figure S4. Percentage of dendritic cells associated with macrophages.**


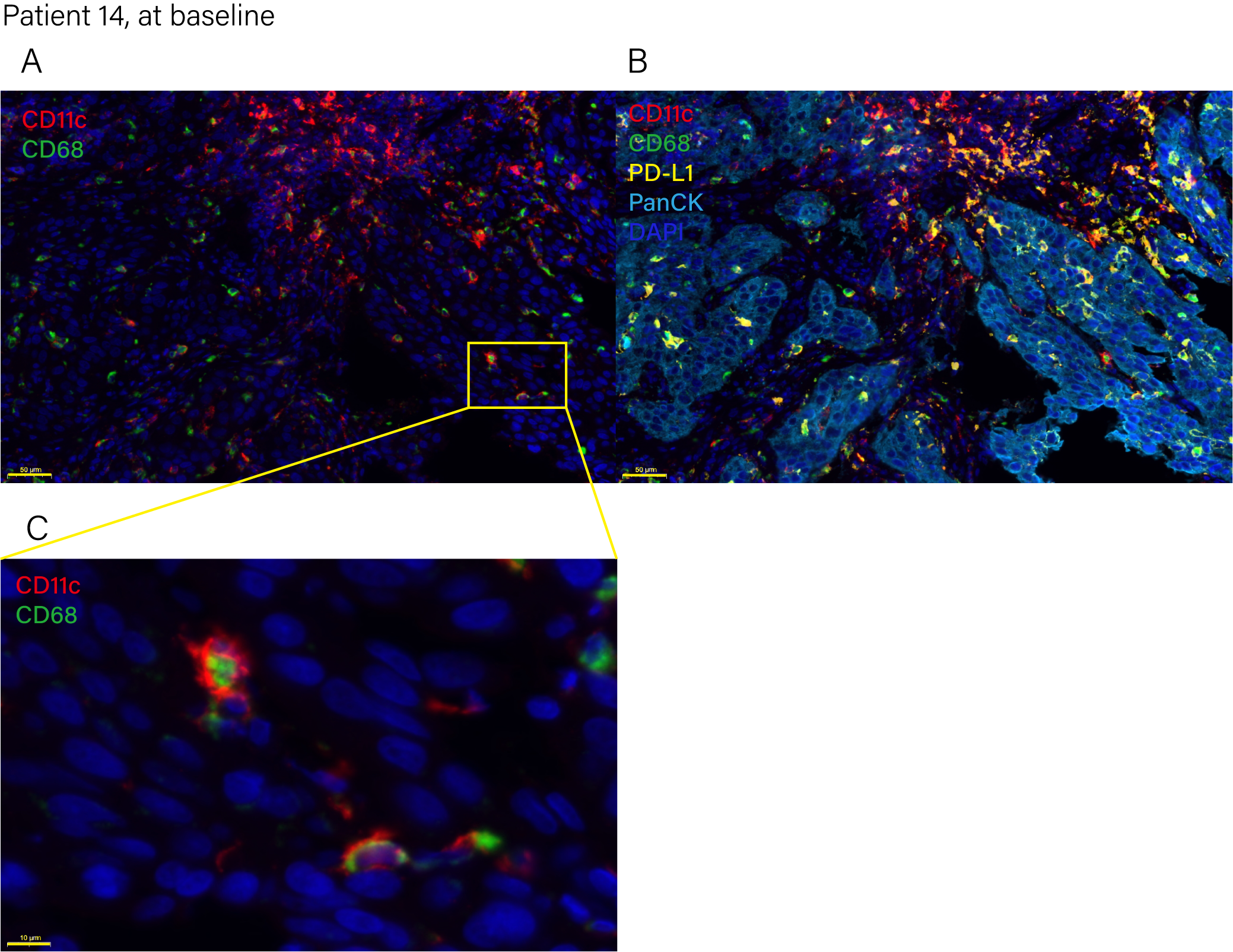


**Figure S5. Low overlap between CD11c and CD68 in ESCC.**

The CD11, CD68, PD-L1 and cytokeratin were stained by using tyramide signal amplification-based multiplex immunofluorescence assay. Rectangle, overlap between CD11c and CD68.
